# Regulation of SLD5 gene expression by miR-370 during acute growth of cancer cells

**DOI:** 10.1038/srep30941

**Published:** 2016-08-08

**Authors:** Keitaro Yamane, Hisamichi Naito, Taku Wakabayashi, Hironori Yoshida, Fumitaka Muramatsu, Tomohiro Iba, Hiroyasu Kidoya, Nobuyuki Takakura

**Affiliations:** 1 Department of Signal Transduction, Research Institute for Microbial Diseases, Osaka University, 3-1 Yamada-oka, Suita, Osaka 565-0871, Japan

## Abstract

SLD5 is a member of the GINS complex, essential for DNA replication in eukaryotes. It has been reported that SLD5 is involved in early embryogenesis in the mouse, and cell cycle progression and genome integrity in Drosophila. SLD5 may be involved in malignant tumor progression, but its relevance in human cancer has not been determined. Here, we found strong SLD5 expression in both human bladder cancer tissues from patients and cell lines. Knockdown of SLD5 using small interfering RNA resulted in reduction of cell growth both *in vitro* and an *in vivo* xenograft model. Moreover, we found that high levels of SLD5 in bladder cancer cells result from downregulation of microRNA (miR)-370 that otherwise suppresses its expression. High level expression of DNA-methyltransferase (DNMT) 1 and IL-6 were also observed in bladder cancer cells. Knockdown of IL-6 led to downregulation of DNMT1 and SLD5 expression, suggesting that IL-6-induced overexpression of DNMT1 suppresses miR-370, resulting in high SLD5 expression. Our findings could contribute to understanding tumorigenic processes and progression of human bladder cancer, whereby inhibition of SLD5 could represent a novel strategy to prevent tumor growth.

In order to carry out nuclear DNA replication during G1 and S phase, many factors are recruited to the chromosomal origin, one of which is the GINS complex composed of SLD5 and partner of Sld5 (Psf) −1, −2, and −3), identified in eukaryotes in 2003^1^. The GINS complex has been reported to regulate DNA polymerase ε (DNA poly ε)^1^. In budding yeast, cyclin-dependent kinases (CDK) activate and load GINS complexes at origins^1^,^2^ to regulate the initiation of DNA replication.

Recently, several studies have shown that each GINS member is associated with malignant progression in several different tumor histotypes, i.e., PSF1 for breast cancer^3^, colon cancer^4^, and lung cancer^5^, PSF2 for cholangiocarcinoma^6^, and PSF3 for colon cancer^7^ and non-small cell lung cancer^8^. Although we previously reported on the levels of expression of SLD5 in different cancer cell lines^7^, to the best of our knowledge there have been no reports thus far on its expression and malignant tumor progression. We did report that targeted disruption of the SLD5 gene led to disturbance of epiblast proliferation and resulted in early embryonic lethality^9^. Moreover, we found that SLD5 is involved in protection from DNA damage in mice^10^. It has also been suggested that SLD5 plays a role in maintaining genome integrity in Drosophila^11^. Therefore, it may be hypothesized that SLD5 has a specific function in tumorigenesis.

Most bladder cancers are non-invasive transitional cell carcinomas, but recurrence and progression rates can be very high. It has been suggested that malignant progression in bladder cancer is associated with chromosomal abnormalities^12^,^13^ and gene mutations in RB1 and p16^13^, TP53^14^, G1 checkpoint protein^15^ and/or cyclin D1^16^,^17^. Apart from these genes, other molecular targets for suppressing tumor progression are likely to exist in bladder cancer.

MicroRNAs (miRNAs) are endogenous RNAs containing approximately 18–25 nucleotides which regulate gene expression and translation by binding to 3′ UTRs of target genes^18^,^19^. So far, over 1000 miRNAs have been discovered, and it has been proposed that around 60% of genes are regulated by miRNAs^20^,^21^. It is widely accepted that changes of miRNA expression in tumors compared with normal tissues affect malignant progression and that such miRNAs may be therapeutic targets. In bladder cancer, several studies have suggested that changes of miRNA expression are involved in tumorigenesis^22^,^23^,^24^.

The aim of the present study was to analyze whether SLD5 expression is associated with tumor growth and if so, how its expression is regulated genetically or epigenetically in tumors, including by miRNAs. Our data suggest that expression of miR-370 is negatively regulated in bladder cancer cells, resulting in upregulation of SLD5 to induce tumor growth. Here, we show how miR-370 is suppressed in association with the functions of IL-6 and DNMT1, and discuss the effectiveness of SLD5 suppression as a potential therapeutic benefit.

## Results

### SLD5 is highly expressed in human bladder cancer tissues

**Figure 1:**
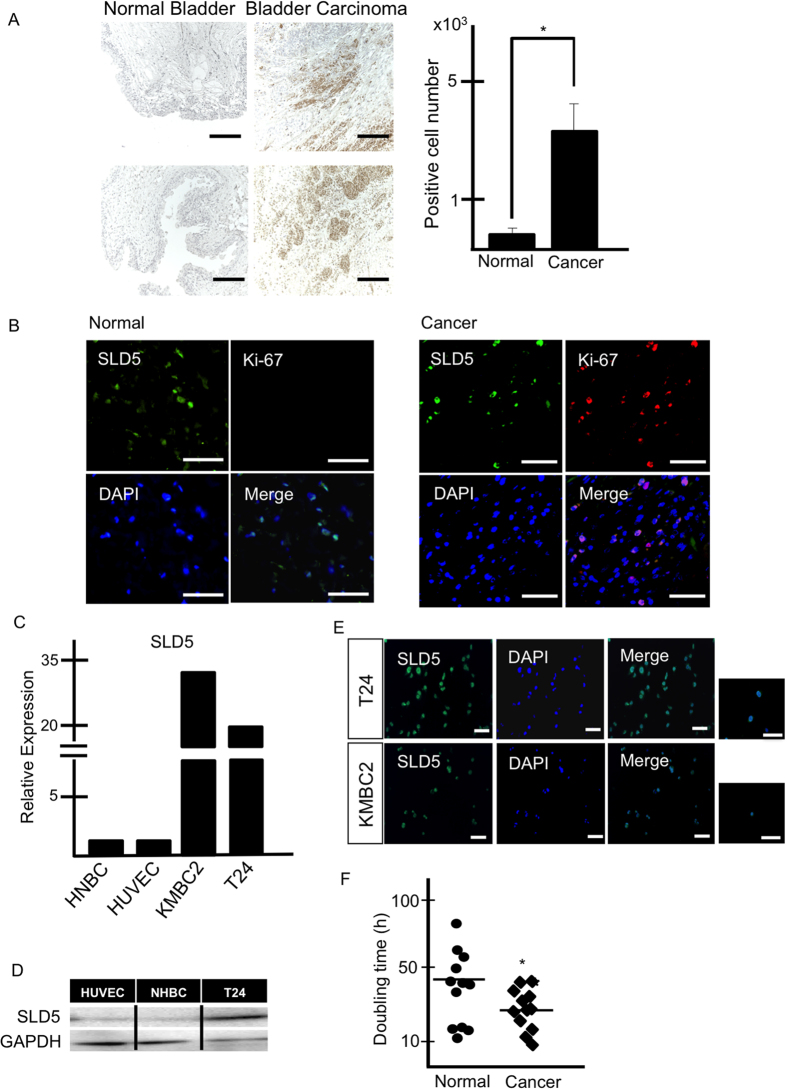
Expression of SLD5 in human bladder cancer tissue. (**A**) Representative images of normal bladder tissue and bladder cancer tissue stained with anti-SLD5 antibody. Samples were counterstained with hematoxylin. Brown: SLD5, Blue: nuclei. Scale bars: 100 μm. Graph shows the quantitative evaluation of SLD5-positive cells in random fields. Data show the mean ± SE, *p < 0.05, (n = 3). (**B**) Immunofluorescence staining of normal and bladder cancer tissues. SLD5 (green), Ki-67 (red), and DAPI (blue). Scale bar: 100 μm. (**C**) Comparison of mRNA expression levels of *SLD5* in normal and cancer cells. Human normal bladder cells (HNBC), human umbilical vein endothelial cells (HUVEC), and human bladder cancer cells (KMBC2, T24) were analyzed by qRT-PCR. Normal cell expression was set at unity. *GAPDH* was used as an internal control. (**D**) Comparison of SLD5 protein expression by Western blotting. HUVECs, HNBC and T24 bladder cancer cells (T24) were used. (**E**) Immunocytochemical analysis of T24 and KMBC2 with anti-SLD5 antibody (green) in cancer cells. Nuclei were stained with DAPI (blue). Scale bars: 100 μm. (**F**) Comparison of doubling time in HUVECs (Normal) and T24 (Cancer). Bars show the mean ± SE, *p < 0.05.

In the tested human cancer arrays, strong SLD5 expression in bladder cancer was found exclusively in transitional cell carcinoma (Fig. 1A). Because SLD5 is a member of the GINS complex, which regulates DNA replication, we asked whether SLD5 expression is related to cell cycle activity and cellular growth. For this, we stained tissues with anti-SLD5 together with anti-Ki-67 antibodies. As shown in Fig. 1B, most of the SLD5-positive cells also expressed Ki-67, suggesting that SLD5 marks the proliferating cancer cells. Some cells in normal bladder tissue also expressed SLD5, but these were Ki-67 negative, suggesting a different role of SLD5 in normal cells.

To assess the expression level of SLD5 in cancer cells relative to normal cells, we analyzed *SLD5* mRNA expression in human bladder cancer cell lines (T24 and KMBC2), in human normal bladder cells (HNBC), and human umbilical vein endothelial cells (HUVECs) using quantitative (q) RT-PCR analysis. Extremely high expression of *SLD5* was observed in cancer cells relative to normal cells (Fig. 1C). We also assessed SLD5 protein expression in bladder cancer cells by Western blotting. As observed for mRNA, bladder cancer cells also expressed higher amounts of SLD5 protein than normal cells (HUVEC and HNBC) (Fig. 1D). Moreover, we confirmed that most of the cultured bladder cancer cells expressed SLD5 protein exclusively in their nuclei (Fig. 1E). T24 cells divided faster than HUVECs (Fig. 1F), suggesting that SLD5 expression may correlate with the rate of cell proliferation and possibly acute tumor growth.

### Knockdown of *SLD5* reduces the number of cells in S phase and Ki-67-positive cells

**Figure 2:**
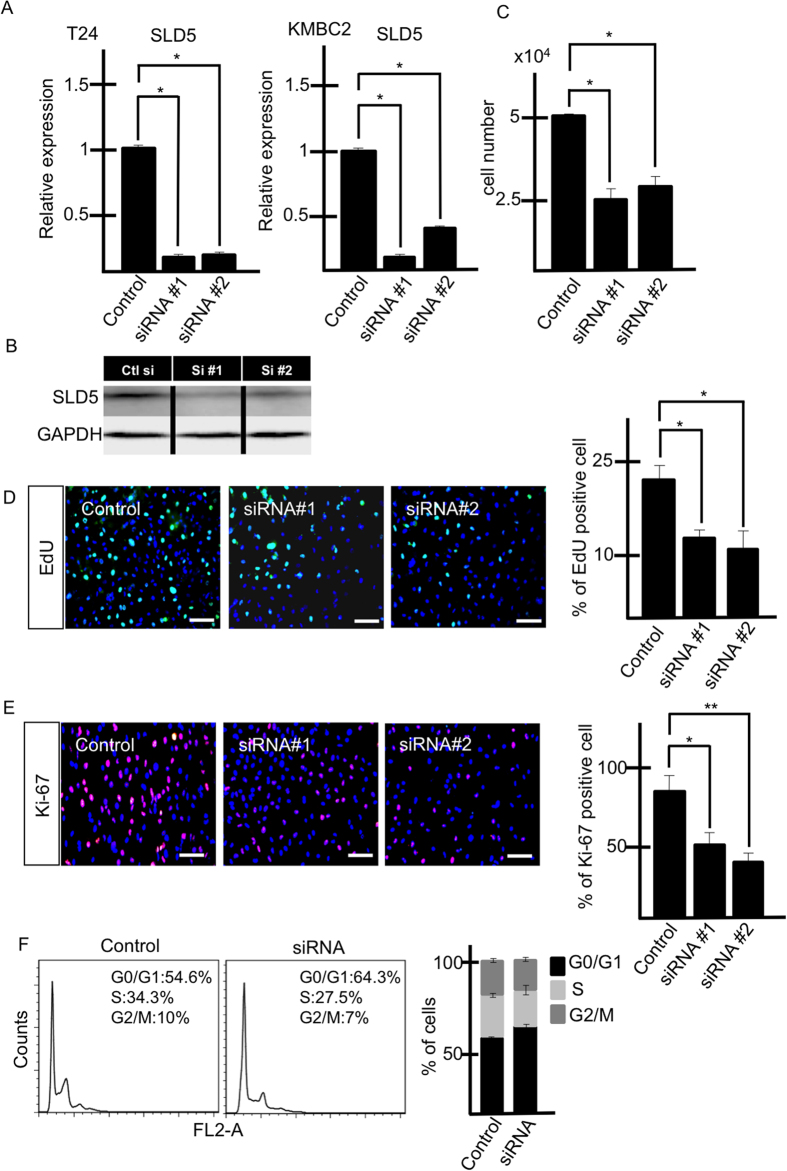
Relevance of SLD5 expression for cell cycle progression. Bladder cancer cell lines (T24, KMBC2) were transfected with two types of SLD5-specific siRNA (#1 and #2). (**A**) Quantitative evaluation of *SLD5* mRNA affected by siRNA. Data are mean ± SE, *p < 0.05 (n = 3). (**B**) Western blotting of SLD5 in control scrambled siRNA (Ctl) and *SLD5*-specific siRNA #1 and #2 treated cells. (**C**) Comparison of cell growth after siRNA transfection (T24). Data are mean ± SE, *p < 0.05 (n = 3). (**D,E**) Cell growth (T24) affected by SLD5 silencing. (**D**) Uptake of EdU (green). Nuclei were stained with Hoechst 33342 (blue). EdU-positive cells were counted and normalized to nuclear numbers, and are shown graphically. Data are mean ± SE, *p < 0.05 (3 random fields) Scale bars: 100 μm. (**E**) Ki-67 staining (red). Nuclei were stained by DAPI (blue). Ki-67-positive cells were counted and normalized to the number of all surviving cells. Results are shown as mean ± SE, *p < 0.05 **p < 0.005 (3 random fields). Scale bars: 100 μm. (**F**) Cell cycle analysis using flow cytometry. After transfection with control or *SLD5*-specific siRNA, cells were analyzed. The right-hand graph depicts the quantitative evaluation by showing mean ± SE (n = 3).

Next, we analyzed whether knocking down *SLD5* affects the growth of bladder cancer cells. For this, we silenced *SLD5* in bladder cancer cells using *SLD5*-specific siRNA. We confirmed the knockdown effect using qRT-PCR and Western blotting (Fig. 2A,B) and found that attenuation of SLD5 expression inhibits the growth of both T24 and KMBC2 cancer cells (Fig. 2C). It is possible that silencing *SLD5* might affect the expression of other GINS members as well, but we found that levels of these were not altered (Supplementary Fig. S1).

When cells were labelled with EdU to analyze DNA synthesis, EdU-positive cells were significantly reduced on silencing *SLD5* (Fig. 2D). Similarly, Ki-67 positivity was reduced by knocking down *SLD5* (Fig. 2E). Cell cycle analysis showed that the fraction of cells in S phase was reduced by knocking down *SLD5* (Fig. 2F). Therefore, we conclude that SLD5 positively regulates the cell cycle of bladder cancer cells.

### Suppression of tumor growth by injection of *SLD5* siRNA *in vivo*

**Figure 3:**
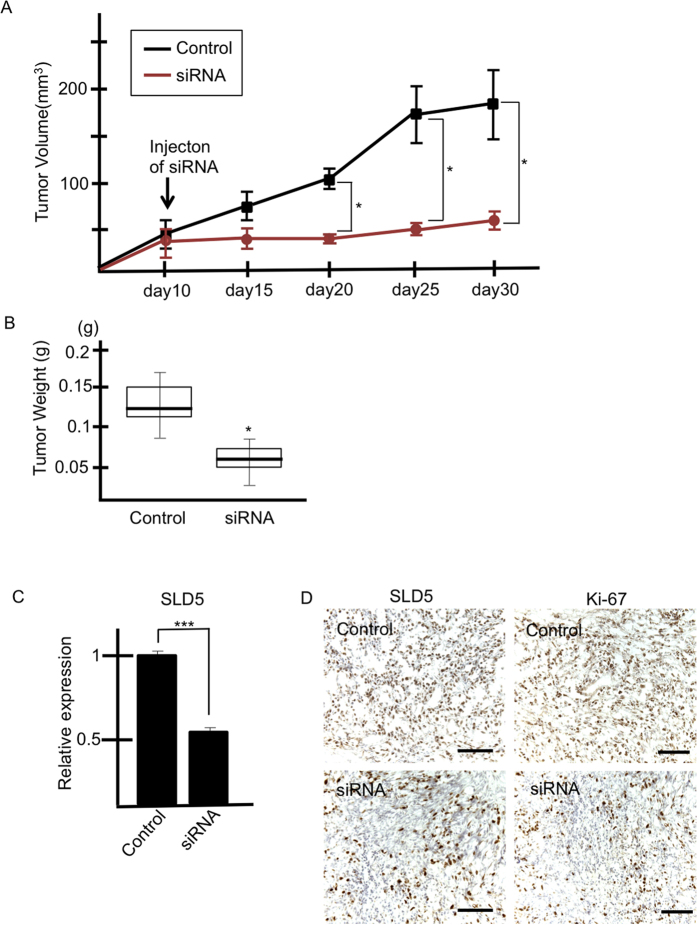
Silencing of SLD5 attenuates tumor growth. Bladder cancer cells (T24) were subcutaneously transplanted into nude mice. Control or *SLD5*-specific siRNA was injected on day10 post-inoculation. (**A**) Time course of tumor volume dynamics. Data are mean ± SE (n = 3). (**B**) Tumor weight on day 30 post-inoculation. *p < 0.05 mean ± SE. (**C**) qRT-PCR analysis for assessment of *SLD5* mRNA expression in control and *SLD5* siRNA-injected tumors. Data are mean ± SE ***p < 0.0001. (**D**) Xenograft tumors injected with control or *SLD5* siRNA were stained with anti-SLD5 or Ki-67 antibody (brown). Sections were counterstained with hematoxylin. Scale bars: 100 μm.

The above *in vitro* experiments had clearly demonstrated that silencing *SLD5* suppresses proliferation of cancer cells. Next, we asked whether silencing of *SLD5* effectively inhibits tumor growth *in vivo*. To assess this, T24 cells were inoculated into nude mice and scrambled (control) or *SLD5*-specific siRNA mixed with atelocollagen was injected into the tumors once they had reached approximately 50 mm^3^ in diameter. After injection of *SLD5*-specific siRNA, tumor growth was clearly suppressed for at least 20 days (Fig. 3A,B).

To confirm reduced expression of *SLD5*, we performed qRT-PCR analysis using RNA from scrambled or *SLD5*-specific siRNA-injected tumors 20 days after treatment. We confirmed that the level of *SLD5* in the tumors injected with specific siRNA was half that of control tumors (Fig. 3C). Moreover, the fraction of SLD5- and Ki-67-double positive cells was also reduced in siRNA-injected tumors, as confirmed by immunohistochemistry (Fig. 3D).

### miR-370 downregulation results in SLD5 upregulation in human bladder cancer cells

**Figure 4:**
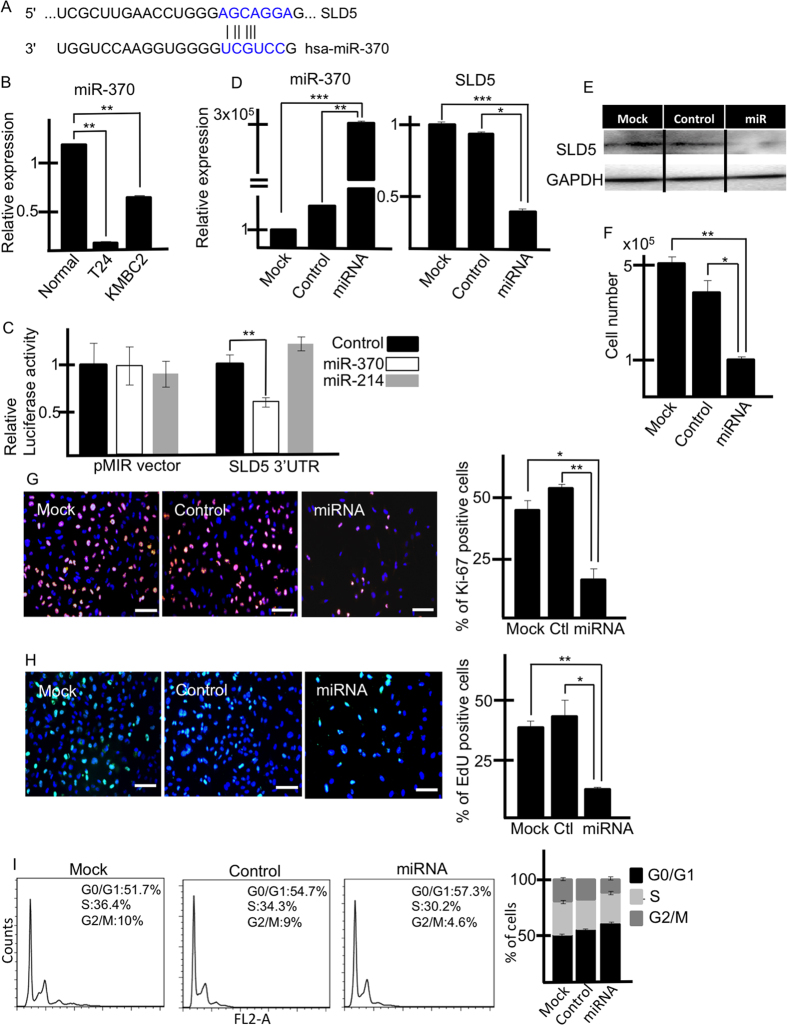
*SLD5* mRNA expression is regulated by miR-370. (**A**) Seed sequence of miR-370 (blue) located in the 3′-UTR of *SLD5*. (**B**) Expression level of miR-370 in human bladder cancer cells compared to HUVECs (Normal). Data are mean ± SE **p < 0.001 (n = 3). The normal cell was set at unity. *U6* is an internal control. (**C**) Luciferase reporter assay. T24 cells were transfected with pMIR luciferase reporter containing 3′-UTR sequences of *SLD5* and miR-370. miR-214 was used as a control. Graph depicts relative luciferase luminescence activity. Control was set at unity. Results are mean ± SE, **p < 0.005 (n = 3). (**D**) Attenuation of *SLD5* expression by miR-370 transfection in T24 cells. The level of miR-370 (left) and *SLD5* (right) after transfection of each miR was confirmed by qRT-PCR. Values for mock control cells were set at unity. *U6* or *GAPDH* was used as an internal control. Data are mean ± SE *p < 0.05. **p < 0.001, ***p < 0.0001. (**E**) Western blotting showing SLD5 expression in miR-370 mimic-transfected T24 cells. (**F**) Cell growth (T24) affected by miR-370 mimic transfection. Data are mean ± SE *p < 0.05. **p < 0.001. (**G**) Ki-67 (red) and (**H**) EdU (green)-positive cells in miRNA-transfected T24 cells. Graphs show quantitative evaluation of Ki-67 or EdU-positive cells. Data are mean ± SE, *p < 0.05,**p < 0.005 (3 random fields). Scale bars: 100 μm. (**I**) Cell cycle analysis using flow cytometry. Cells were analyzed after transfection with control or miR-370 mimic. The right-hand graph depicts a quantitative evaluation by showing mean ± SE (n = 3).

SLD5 expression is lower in normal cells than in bladder cancer cells (Fig. 1). This suggests that miRNAs negatively regulating SLD5 expression are downregulated in cancer cells. To identify candidate miRNAs responsible for this, we sought those which interact with the 3′-UTR region of the *SLD5* gene using Targetscan, as in previous reports showing an association with bladder cancers^22^,^23^,^24^. Among several candidates, we found that a miR-370 target sequence is located at the 3′-UTR of the *SLD5* gene. We confirmed that miR-370 expression was indeed suppressed in T24 and KMBC2 cells (Fig. 4A,B).

To document direct binding of miR-370 to the 3′-UTR of the *SLD5* gene, we performed luciferase reporter assays. T24 cancer cells were transfected with pMIR luciferase vector with an inserted 3′-UTR region of *SLD5* containing the miR-370 target sequence. Addition of a miR-370 mimic led to reduction of the luciferase activity of the *SLD5* 3′-UTR, whereas miR-214 as a negative control had no affect (Fig. 4C). Therefore, we conclude that miR-370 directly binds to the 3′-UTR of the *SLD5* gene.

Next, we investigated how miR-370 affects SLD5 expression. We overexpressed it in T24 bladder cancer cells using miRNA mimic transfection methods, and assessed *SLD5* mRNA and SLD5 protein levels using qRT-PCR (Fig. 4D) and Western blotting (Fig. 4E), respectively, after 48 hours. The results suggested that miR-370 inhibits mRNA expression in T24 cells, resulting in a reduction of SLD5 protein expression.

Next, we investigated whether miR-370 silencing affects SLD5 expression in normal cells. We knocked down miR-370 in normal cell lines (HUVEC) using miR-370 inhibitor transfection methods and assessed *SLD5* mRNA and SLD5 protein levels using qRT-PCR and Western blotting, respectively (Supplementary Fig. 3). The results suggested that inhibition of miR-370 led to increased *SLD5* mRNA and protein expression in normal cells.

As observed in the siRNA experiments (Fig. 2), miR-370 overexpression also inhibited the proliferation of bladder cancer cells (Fig. 4F). Moreover, we analyzed cell kinetics and how the cell cycle was affected by miR-370. As shown in Fig. 4G,H, the fraction of Ki-67- and EdU-positive cells was reduced in miR-370-transfected cells compared to controls. In terms of the cell cycle, similar to the results from the *SLD5* knockdown study, miR-370 mimic transfection reduced the fraction of cells in S phase relative to control miR transfection (Fig. 4I).

### A miRNA-370 mimic inhibits tumor growth *in vivo*

**Figure 5:**
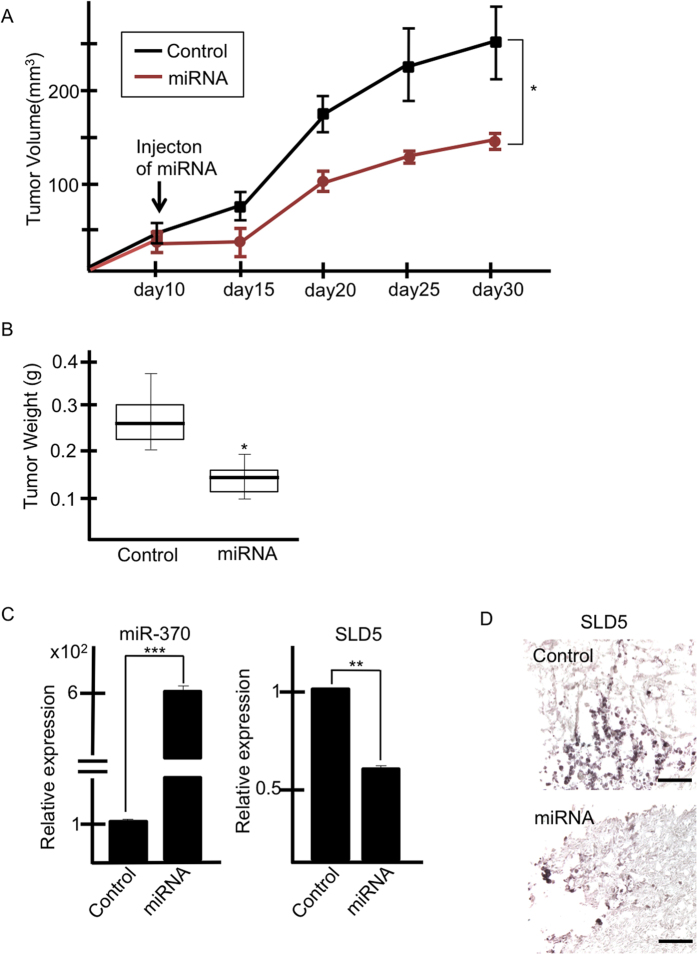
Tumor growth is affected by miR-370. (**A,B**) Bladder cancer cells (T24) were subcutaneously transplanted into nude mice. miR-370 mimics or control miR were injected on day10 post-inoculation. (**A**) Time course of tumor volume dynamics. Data are mean ± SE (n = 3). (**B**) Tumor weight on day 30 post-inoculation. *p < 0.05 mean ± SE (**C**) qRT-PCR analysis for assessment of miR-370 or *SLD5* mRNA expression in tumors injected with control or miR-370. Data are mean ± SE, **p < 0.0005, ***p < 0.0001 (n = 3). (**D**) Xenografted tumors injected with control or miR-370 mimics were stained with anti-SLD5 antibody (brown). Sections were counterstained with hematoxylin. Scale bars: 100 μm.

*In vitro* experiments had clearly demonstrated that overexpression of miR-370 suppresses proliferation of cancer cells. Next, we investigated whether miR-370 can effectively inhibit tumor growth *in vivo*. To assess this, T24 cells were inoculated into nude mice and control miR or miR-370 mixed with atelocollagen was injected into the tumors once they had reached approximately 50 mm^3^ in diameter. After injection of miR-370, tumor growth was clearly suppressed (Fig. 5A,B).

We assessed the level of expression of miR-370 and *SLD5* by qRT-PCR using tumor tissue on day 20 after treatment, and confirmed overexpression of miR-370 and downregulation of *SLD5* in tumors from the miR-370-injected group. Moreover, we confirmed that SLD5 protein was also suppressed in tumor tissue of miR-370-injected relative to control tumors (Fig. 5C,D).

### Regulation of miR-370 in human bladder cancer cells

**Figure 6:**
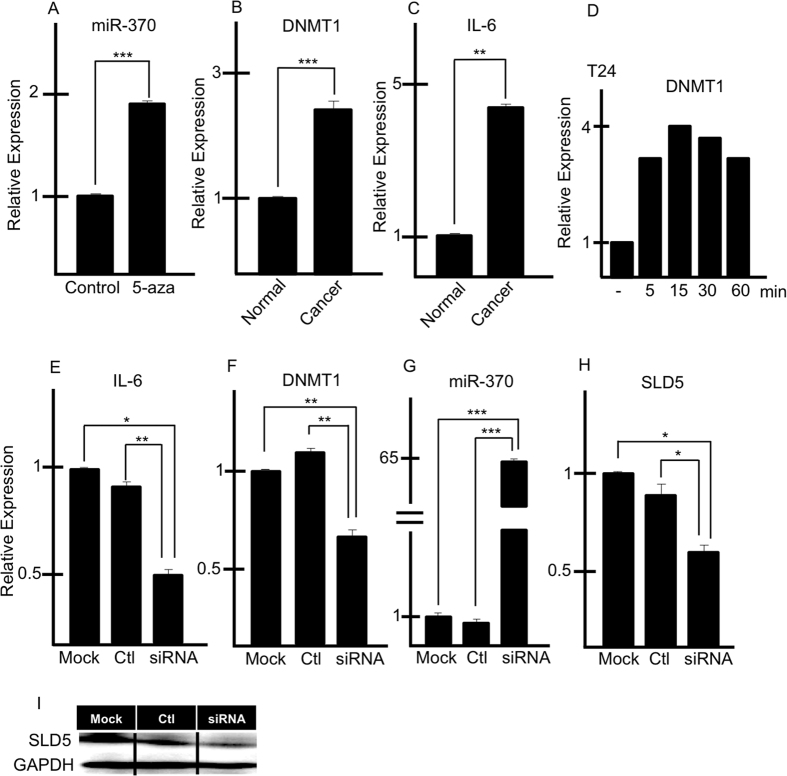
Regulation of miR-370 expression in tumors. (**A**) miR-370 expression following 5-azacitidine treatment of T24 cells. Results are mean ± SE ***p < 0.0005 (n = 3). (**B,C**) qRT-PCR analysis of *DNMT1* (**B**) and *IL-6* (**C**) mRNA expression in normal bladder cells (Normal) and a cancer cell line (T24; Cancer). Data are mean ± SE, **p < 0.0005, *** < 0.00001 (n = 3). (**D**) qRT-PCR analysis of *DNMT1* mRNA expression in T24 cells after treatment with IL-6. (**E–H**) qRT-PCR analysis of *IL-6* (**E**), *DNMT1* (**F**), miR-370 (**G**) and *SLD5* (**I**) expression in T24 cells 48 hours after transfection of *IL-6*-specific siRNA or control siRNA (ctl). Data are mean ± SE, *p < 0.05, **p < 0.005, ***p < 0.00001 (n = 3). (**I**) Western blotting for detection of SLD5 in cells as described in (**E**).

It has been reported that the expression of miRNAs is regulated by chromosomal modifications^25^. We analyzed whether miR-370 expression is regulated by DNA methylation by using 5-azacytidine (5-aza), an inhibitor of DNA methylation. After 24 hours of treatment with 5-aza, miR-370 expression was more strongly induced in cancer cells than in control cells (Fig. 6A), suggesting that its expression is suppressed in cancer cells by DNA methylation.

Next, we analyzed DNA methylation-related gene expression by qRT-PCR (Fig. 6B). Of the three DNA methyltransferases (DNMTs) in mammals, i.e., DNMT1, DNMT3a, and DNMT3b, DNMT1 is the most abundant in adult cells^26^. We found that T24 bladder cancer cells express two-fold higher levels of *DNMT1* than normal cells (Fig. 6B). It has been reported that IL-6 expression correlates with DNMT1 expression^27^. Accordingly, we found that *IL-6* expression is also upregulated in bladder cancer cells (Fig. 6C). When T24 cells were stimulated with IL-6, *DNMT1* expression was induced (Fig. 6D), suggesting that an autocrine loop of IL-6 in T24 cells upregulates *DNMT1*. Consistent with this, knocking down *IL-6* using siRNA resulted in reduced *DNMT1* expression (Fig. 6E,F) and concurrent upregulation of miR-370 (Fig. 6G) and reduction of *SLD5* (Fig. 6H). We confirmed that SLD5 expression was suppressed not only at the RNA but also at the protein level (Fig. 6I). Therefore, we conclude that in bladder cancer cells, up-regulation of IL-6 induces SLD5 expression to promote acute proliferation of cancer cells.

## Discussion

In this study, we document pivotal roles of SLD5 in acute proliferation of human bladder cancer cells. In normal cells, SLD5 expression is blocked by miR-370 expression. However, in cancer cells, IL-6 induces DNMT1 to inhibit miR-370 expression. Because miR-370 suppresses SLD5, in cancer cells SLD5 expression is restored and cells can proliferate. SLD5 is a member of the GINS complex, acting on DNA replication^1^,^18^. We previously reported that SLD5 is essential for early embryogenesis and that SLD5-knockout mice were embryonic lethal because of impaired proliferation of cells in the inner cell mass^7^. In terms of SLD5 expression in tumors, a few papers have indicated its overexpression, including in breast cancer^3^ and colorectal cancer^28^. Here, we report the overexpression of SLD5 in bladder cancer cells. We analyzed the expression of SLD5 and its relationship to prognosis of bladder cancer patients (n = 165) by using public database PrognoScan (http://www.abren.net/PrognoScan/). Overall survival rate in that research indicated that SLD5 expression was significantly associated with poor survival rate (p = 0.013). Therefore, our basic research contains clinical implication.

In S phase, cell division cycle (cdc) 45, mini chromosome maintenance protein (MCM) 2–7 and GINS complex cooperate as a replicative helicase CMG complex^29^. Especially, GINS is essential for the maintenance of interaction cdc45 and MCM2-7. Because GINS contributes to activity of CMG complex, increasing expression level of SLD5, a member of GINS complex might leads to aberrant proliferation in cancer cells. Moreover, tumor suppressor gene, Rb1, works as an S phase transition inhibitor is often mutated in bladder cancer patients^30^. Because of this reason, it is possible that Rb1 mutation also leads to SLD5 overexpression in bladder cancer cells.

Regarding other members of the GINS complex, PSF1 was also overexpressed along with SLD5 in bladder cancer cells, but PSF2 and PSF3 levels were similar to those in normal cells such as HUVECs (data not shown). This expression profile in bladder cancer cells is similar to that reported in breast cancer cells^3^. As it has been suggested that psf2 and psf3 act as multicopy suppressors of sld5 and psf1 in budding yeast^1^, our results suggest that SLD5 is critical for proliferation of cancer cells, and therefore that PSF2 and PSF3 overexpression in cancer cells may not be required.

According to our results, expression of SLD5 is significantly upregulated both in human bladder cancer tissue samples as well as in cell lines. Moreover, decreased cell numbers, reduced fractions of cells in S phase, and suppressed tumor growth were observed following silencing of *SLD5*. Furthermore, we identified miR-370 as a negative regulator of *SLD5* gene expression. Many studies showing associations of miR with tumorigenesis have been reported in bladder cancer. In bladder cancer cells, miR-101, −203, and −204^22^,^23^,^24^ are downregulated, resulting in induction of aberrant increases of proliferative capacity or cell migration. Although several lines of evidence indicate downregulation of miR-370 in cancer cells^23^,^31^,^32^, the relationship between miR-370 and SLD5 has not been reported previously. Based on our results, we hypothesize that miR-370 acts as a negative regulator of SLD5 expression in normal states. In contrast, in bladder cancer cells, miR-370 is downregulated, and SLD5 expression levels are increased to induce proliferation of cancer cells. In Fig. 4, overexpression of miR-370 might affect not only cancer cells but other cells consisting cancer microenvironment. Because endothelial cells, blood cells (data not shown) and fibroblastic cells^33^ express GINS complex gene, reduced SLD5 expression by the injection of miR-370 caused attenuation of cellular activity of those cells and cancer cell proliferation and survival could not be supported by such stromal cells.

We found that attenuated miR-370 expression in cancer cells is partly mediated by methylation of the miR-370 promoter, because treatment with 5-aza-2-deoxycytidine (5-aza), a specific inhibitor of DNA methylation^34^, induced the expression of this miRNA. Therefore, bladder cancer cells may have intrinsic signaling pathways for methylating the miR-370 promoter region. So far, many studies have focused on IL-6 as a player in malignant tumor progression by facilitating epithelial-mesenchymal transition (EMT), inflammation, and angiogenesis. Moreover, it has been reported that IL-6 is involved in DNMT1 expression^27^,^35^,^36^. Here, we also found that IL-6 and DNMT1 expression were correlated in bladder cancer. It is possible that upregulated DNMT1 leads to hypermethylation of CpG islands in the promoter regions of many genes in bladder cancer cells^37^. In addition to DNMT1 expression, we found that the endogenous demethylation factor, ten-eleven translocation family methylcytosine hydroxylase (TET1)^38^ was markedly lower in cancer cells (Supplementary Fig. S2). These results suggest that hypermethylation caused by IL-6 induces suppression of many genes including miR-370, contributing to the upregulation of SLD5. It has also been suggested that miR-370 is normally imprinted and activated only on the maternal allele^35^, so it may be predicted that miR-370 is downregulated even when CpG islands are only slightly methylated.

In summary, to the best of our knowledge, there are no published studies focused on the behavior of SLD5 in human cancer cells. The present study revealed a relationship between SLD5 and miR-370 in human bladder cancer. Although we focused on miR-370, it is possible that there are multiple additional miRNAs that are relevant for SLD5 expression *in vivo*. It has been reported that not only SLD5 but also other GINS members have the potential to regulate cancer proliferation and that these genes might be cancer therapeutic targets. Further studies on the regulation of SLD5 expression are required to develop this moiety as a drug target.

## Methods

### Cell lines and samples

Human bladder cancer cell lines T24 and KMBC2 (JCRB, Osaka, Japan) were maintained in MEM medium (Sigma, St. Louis, MO) and Ham’s F12 medium (GIBCO, Rockville, MD), respectively, with 10% fetal bovine serum (FBS; Sigma, St. Louis, MO) and penicillin/streptomycin (GIBCO, Rockville, MD). Human umbilical vein endothelial cells (HUVECs KURABO, Osaka, Japan) were maintained in HuMedia EG2 (KURABO, Osaka, Japan). For qRT-PCR analysis, normal human bladder RNA sample was purchased from US Biomax (Rockville, MD).

### Immunohistochemistry

Human carcinoma tissue array specimens and bladder cancer tissue specimens including normal human bladder were purchased from Biochain (Beijing, China) and US Biomax (Rockville, MD), respectively. For immunohistochemical analysis, after deparaffinization, sections were activated with citrate buffer solution. Blocking with skimmed milk in PBST (0.1% TritonX-100 in PBS) for 1 h. Anti-SLD5 antibody (1:200 Iwaki, Japan) was used as the primary antibody with biotin-conjugated goat anti-rat IgG (1:200, Jackson ImmunoResearch, West Grove, PA) as the secondary antibody. Avidin-biotin complex (ABC) kits (Vector Laboratories, Burlingame, CA) and 3,3′-diaminobenzidine (DAB; Dojindo, Japan) and nickel chloride (NiCl_2_, Wako, Japan) were used for visualization. For staining of nuclear DNA, hematoxylin (Wako) was applied in counterstaining.

For the fluorescent immunohistochemical analysis, rat anti-SLD5 antibody (1:200; Iwaki), rabbit anti-SLD5 (1:200 Abcam, Cambridge, UK), rat anti-CD44-PE (1:100 eBioscience), and mouse anti-Ki-67 (1:400 Dako, Carpentaria, CA) were used as primary antibodies and donkey-anti-rat IgG Alexa Fluor 488 (1:200, Jackson ImmunoResearch) and goat anti-mouse IgG Alexa Fluor 546 (1:200, Jackson ImmunoResearch) as secondary antibodies. Nuclear DNA was counterstained with Hoechst 33342 (1:3000, Sigma) or DAPI (1:3000 Dojindo,). Stained samples were assessed under a microscope (CTR5500, Leica, Wetzlar, Germany). Images were analyzed using Image J software (NIH).

### Immunocytochemistry

Cells were fixed with 4% paraformaldehyde for 10 min and blocked with skimmed milk in PBST (0.1% Triton X-100 in PBS) for 1 h. Cells were then incubated with rabbit anti-SLD5 (1:200) or mouse anti-Ki-67 (1:200) overnight at 4 °C, washed with PBS, and then incubated with donkey anti-rabbit Alexa Fluor 488 (1:200, Jackson ImmunoResearch) or goat anti-mouse Alexa Flour546 (1:200, Jackson ImmunoResearch) for 1 h at room temperature. Nuclear DNA was counterstained with DAPI (1:3000 Dojindo). Stained samples were assessed under a microscope and images were analyzed using ImageJ software.

### Quantitative Reverse Transcription-PCR

Total RNA was isolated from cells or FFPE sections using the RNeasy plus mini kit (Qiagen, Chatsworth, CA) and the Nucleospin total RNA FFPE (MACHEREY-NAGEL, Germany), respectively, according to the manufacturer’s protocol. Total RNAs were reverse transcribed using the PrimeScript RT reagent kit (Takara bio, Shiga, Japan). Quantitative RT-PCR (qRT-PCR) was performed using SYBR Premix Ex Taq II (Takara) on Mx3000P (Stratagene, La Jolla, CA) as previously described^39^. Baseline and threshold were adjusted according to the manufacturer’s protocol. Levels of the specific amplified genes were normalized to the level of GAPDH. All primers are listed in Supplementary Table 1 and were purchased from GeneDesign, Inc (Osaka, Japan).

For detection of miRNA expression, total and small RNAs were extracted using NucleoSpin miRNA kits (Takara clontech) followed by small RNA reverse transcription using Mir-X miRNA First-Strand Synthesis Kits (Takara clontech). The qRT-PCR reaction was performed according to the manufacturer’s protocol using specific primers (Supplementary Table 1). The 2^−ΔΔCt^ method was used to calculate results and amplified genes were normalized to the level of U6.

### RNA interference

SLD5 and IL-6 expression in human bladder cancer cell lines was transiently knocked down with small interfering RNA (siRNA) (Invitrogen, Carlsbad, CA or Thermofisher Scientific Inc, Massachusetts, respectively). Lipofectamine 2000 (Invitrogen) was used for the transfection of siRNA, following the manufacturer’s instructions. We used two different siRNA oligonucleotides for SLD5 and scrambled siRNA (Invitrogen). The Pre-hsa-miR-370 mimic (GeneDesign, Osaka, Japan) was transfected into T24 bladder cancer cells following the manufacturer’s instructions.

### Western blotting

Total cells were lysed using radioimmunoprecipitation assay (RIPA) lysis buffer. Normal human bladder whole cell lysate was purchased from Abcam. Proteins were heated for 3 min at 95 °C, loaded onto SDS-polyacrylamide gels and then transferred onto polyvinylidene difluoride membranes (Millipore, Jaffery, NH). Blocking was with 3% BSA prior to staining with anti-SLD5 antibody (1:500 Abcam) or anti-GAPDH (1:3000; Millipore) as primary antibodies overnight at 4 °C. Blots were developed with anti-rabbit HRP and anti-mouse HRP antibodies (Jackson ImmunoResearch) using enhanced chemiluminescence (Pierce Biotechnology Inc, Rockford). Images were analyzed with ImageJ software.

### Measurement of doubling time

To calculate cell growth rate, bladder cancer cell (T24) and normal cells (HUVEC) were cultured for 6, 12, 24 or 48 hours. Cells were counted and numbers were calculated using the following formula: duration × log2/log (final cell number) − log (initial cell number).

### Flow cytometry and EdU incorporation assay

To analyze DNA content, cells were fixed in 70% ethanol and suspended in propidium iodide (PI)/RNase/Triton X-100 staining solution, and then analyzed by FACScalibur (BD Biosciences). Cells were plated into 24-well plates and transfected with siRNA and miRNA. After transfection, EdU incorporated into cells was visualized using Click-iT EdU Alexa Fluor 488 imaging kits (Invitrogen, Carlsbad, CA, USA), according to the manufacturer’s instructions.

### Analysis of xenografted tumor with siRNA and miRNA *in vivo* transfection

2 × 10^6^ T24 cells suspended in 100 μl of phosphate-buffered saline (PBS) were subcutaneously injected into the dorsal surfaces of 6-week-old female KSN/Slc nude mice (Slc, Shizuoka, Japan). Two weeks after transplantation, the developed tumors were treated with control scrambled RNA, SLD5 siRNA, or miR-370 mimic suspended in atelocollagen (Atelogene, KOKEN, Tokyo, Japan) to facilitate siRNA introduction into tumors, according to the manufacturer’s instructions Twenty days after atelocollagen injection, xenograft tumor volumes and weights were measured, RNA was isolated from tumors for the analysis of SLD5 or miR-370 expression by qRT-PCR, and tumor tissues were fixed in 4% paraformaldehyde for immunohistochemistry analysis. Tumor volumes were calculated using the following formula: (major axis) × (minor axis)^2^ × 0.5. All animal experiments were approved by the Animal Research Committee of Osaka University. All experiments were carried out under the guidelines of Osaka University Committee for animal and recombinant DNA experiments.

### Luciferase reporter assay

The 3′ UTR region of SLD5, containing miR-370 target sites, was amplified using a standard PCR protocol with the following linker primers: 5′-ATT ACT ACT AGT GCA TAA ACA GCC AGG CAT GGT GAC-3′ (forward), 5′-AAC CAT AAG CTT TAG TAG AGA TGG GTT TAG TAG AG-3′ (reverse). cDNA was inserted into SpeI and HindII sites of pMIR-REPORT Luciferase vectors (Applied Biosystems, Carlsbad). T24 bladder cancer cells were plated in 96-well plates and transfected with or without miR-370 mimic and luciferase vector. After transfection, luciferase reporter activity in the cells was measured using the Dual-Glo Luciferase Assay System (Promega, Madison, WI). We used the pRL-TK vector (Promega) for normalized values of reporter activity, and a miR-214 mimic was used as a negative control. All transfections were performed using Lipofectamine 2000 (Invitrogen) according to the manufacturer’s instructions.

### DNA demethylation

T24 cells were treated with 1 μM 5-aza-2-deoxycytidine (Sigma Aldrich). After 24 h, miRNA was isolated and cDNA synthesized for qRT-PCR analysis of miR-370. Levels of the specific amplified genes were normalized to the level of U6.

### hIL-6 treatment in a normal cell line (HUVEC) and a cancer cell line (T24)

Normal and cancer cell lines were treated with 100 ng/ml human recombinant IL-6 (Biolegend, San Diego, CA). RNA from recovered cells after treatment was isolated for qRT-PCR analysis of DNMT1. Levels of the specific amplified genes were normalized to the level of GAPDH.

### Statistical analysis

Results are expressed as the mean ± standard error (SE). Student’s t-test was used for statistical analysis. Differences were considered statistically significant if the p-value was less than 0.05.

## Additional Information

**How to cite this article**: Yamane, K. *et al*. Regulation of SLD5 gene expression by miR-370 during acute growth of cancer cells. *Sci. Rep*. **6**, 30941; doi: 10.1038/srep30941 (2016).

## Supplementary Material

Supplementary Information

## References

[b1] Takayama Y. . GINS, a novel multiprotein complex required for chromosomal DNA replication in budding yeast. Genes Dev. 17, 1153–1165 (2003). 12730134 10.1101/gad.1065903 PMC196052

[b2] Yabuuchi H. . Ordered assembly of Sld3, GINS and Cdc45 is distinctly regulated by DDK and CDK for activation of replication origins. EMBO J. 25, 4663–4674 (2006). 16990792 10.1038/sj.emboj.7601347 PMC1589995

[b3] Nakahara I. . Up-regulation of PSF1 promotes the growth of breast cancer cells. Genes Cells. 15, 1015–1024 (2010). 20825491 10.1111/j.1365-2443.2010.01442.x

[b4] Wen J. Z. . Expression of PSF1 in colon cancer tissues and its effect on the proliferation of colon cancer cells. Zhonghua Wei Chang Wai Ke Za Zhi. 16, 70–74 (2013). 23355245

[b5] Zhang J. . Knockdown of PSF1 expression inhibits cell proliferation in lung cancer cells *in vitro*. Tumour Biol. 36, 2163–2168 (2015). 25398693 10.1007/s13277-014-2826-8

[b6] Obama K. . Up-regulation of PSF2, a member of the GINS multiprotein complex, in intrahepatic cholangiocarcinoma. Oncol Rep. 14, 701–706 (2005). 16077978

[b7] Nagahama Y. . PSF3 marks malignant colon cancer and has a role in cancer cell proliferation. Biochem Biophys Res Commun. 392, 150–154 (2010). 20059967 10.1016/j.bbrc.2009.12.174

[b8] Tane S. . Significant role of Psf3 expression in non-small-cell lung cancer. Cancer Sci. 106, 1625–1634 (2015). 26291987 10.1111/cas.12770 PMC4714687

[b9] Mohri T. . Requirement of SLD5 for early embryogenesis.2013, PLoS One. 8, e78961 (2013). 24244394 10.1371/journal.pone.0078961 PMC3823970

[b10] Gong Z. Y. . DNA damage enhanced by the attenuation of SLD5 delays cell cycle restoration in normal cells but not in cancer cells. PLoS One. 9, e110483 (2014). 25334017 10.1371/journal.pone.0110483 PMC4204874

[b11] Gouge C. A. & Christensen T. W. Drosophila Sld5 is essential for normal cell cycle progression and maintenance of genomic integrity. Biochem Biophys Res Commun. 400, 145–150 (2010). 20709026 10.1016/j.bbrc.2010.08.033 PMC2939264

[b12] Cairns P. . Initiation of bladder cancer may involve deletion of a tumour-suppressor gene on chromosome 9. Oncogene 8, 1083–1085 (1993). 8096074

[b13] Hazra A. . Benzoapyrene diol epoxide-induced 9p21 aberrations associated with genetic predisposition to bladder cancer. Genes Chromosomes Cancer. 41, 330–338 (2004). 15390186 10.1002/gcc.20093

[b14] Williamson M. P. . The spectrum of TP53 mutations in bladder carcinoma. Genes Chromosomes Cancer. 9, 108–118 (1994). 7513540 10.1002/gcc.2870090206

[b15] Sheu Y. J. & Stillman B. The Dbf4-Cdc7 kinase promotes S phase by alleviating an inhibitory activity in Mcm4. Nature. 463, 113–117 (2010). 20054399 10.1038/nature08647 PMC2805463

[b16] El-Gamal E. M. & Gouida M. S. Flow cytometric study of cell cycle and DNA ploidy in bilharzial bladder cancer. Clin Lab. 61, 211–218 (2015). 25974985 10.7754/clin.lab.2014.140609

[b17] Eissa S. . HER2/neu expression in bladder cancer: relationship to cell cycle kinetics. Clin Biochem. 38, 142–148 (2005). 15642276 10.1016/j.clinbiochem.2004.09.004

[b18] Bartel D. P. MicroRNAs: genomics, biogenesis, mechanism, and function. Cell. 116, 281–297 (2004). 14744438 10.1016/s0092-8674(04)00045-5

[b19] Lee R. C. . The C. elegans heterochronic gene lin-4 encodes small RNAs with antisense complementarity to lin-14. Cell. 75, 843–854 (1993). 8252621 10.1016/0092-8674(93)90529-y

[b20] Friedman R. C. . Most mammalian mRNAs are conserved targets of microRNAs. Genome Res. 19, 92–105 (2009). 18955434 10.1101/gr.082701.108 PMC2612969

[b21] Friedländer M. R. . Evidence for the biogenesis of more than 1,000 novel human microRNAs. Genome Biol. 15, R57 (2014). 24708865 10.1186/gb-2014-15-4-r57 PMC4054668

[b22] Bo J. . microRNA-203 suppresses bladder cancer development by repressing bcl-w expression. FEBS J. 278, 786–792 (2011). 21205209 10.1111/j.1742-4658.2010.07997.x

[b23] Yoshino H. . The tumour-suppressive function of miR-1 and miR-133a targeting TAGLN2 in bladder cancer. Br J Cancer. 104, 808–818 (2011). 21304530 10.1038/bjc.2011.23 PMC3048214

[b24] Hu Z. . MicroRNA-101 suppresses motility of bladder cancer cells by targeting c-Met. Biochem Biophys Res Commun. 435, 82–87 (2013). 23618864 10.1016/j.bbrc.2013.04.042

[b25] Jody C. . Epigenetics and microRNAs. Pediatric Research 61, 24R–29R (2007). 10.1203/pdr.0b013e3180457684 17413852

[b26] Araujo F. D. . Identification of initiation sites for DNA replication in the human dnmt1 (DNA-methyltransferase) locus. J Biol Chem. 274, 9335–9341 (1999). 10092611 10.1074/jbc.274.14.9335

[b27] Chen M. F. . IL-6 expression regulates tumorigenicity and correlates with prognosis in bladder cancer. PLosOne. 8, e61904 (2013). 10.1371/journal.pone.0061901 PMC3640078 23637926

[b28] Pillaire M. J. . A ‘DNA replication’ signature of progression and negative outcome in colorectal cancer. Oncogene. 29, 876–887 (2010). 19901968 10.1038/onc.2009.378

[b29] Karim Labib and Agnieszka Gambus. A key role for the GINS complex at DNA replication forks. TREND in Cell Biology. Review 17, 271–278 (2007). 10.1016/j.tcb.2007.04.002 17467990

[b30] Sourvinos G. . Genetic detection of bladder cancer by microsatellite analysis of p16, RB1 and p53 tumor suppressor genes. J Urol. 165 249–252 (2001). 11125419 10.1097/00005392-200101000-00073

[b31] Wang C. . Up-regulation of p21 (WAF1/CIP1) by miRNAs and its implications in bladder cancer cells. FEBS Lett. 588, 4654–4664 (2014). 25447520 10.1016/j.febslet.2014.10.037

[b32] Wang C. . Promoter-associated endogenous and exogenous small RNAs suppress human bladder cancer cell metastasis by activating p21 (CIP1/WAF1) expression. Tumour Biol. doi: 10.1007/s13277-015-4571-z (2015). 26643891

[b33] Kong L. . Identification and characterization of mouse PSF1-binding protein SLD5. Biochem Biophys Res Commun. 339, 1204–1207 (2006). 16338220 10.1016/j.bbrc.2005.11.136

[b34] Saito Y. . Specific activation of microRNA-127 with down-regulation of the proto-oncogene BCL6 by chromatin-modifying drugs in human cancer cells. Cancer Cell. 9, 435–443 (2006). 16766263 10.1016/j.ccr.2006.04.020

[b35] An F. . Silencing of miR-370 in human cholangiocarcinoma by allelic loss and interleukin-6 induced maternal to paternal epigenotype switch. PLoS One. 7, e45606 (2012). 23110045 10.1371/journal.pone.0045606 PMC3478287

[b36] Meng F. . Epigenetic regulation of microRNA-370 by interleukin-6 in malignant human cholangiocytes. Oncogene. 27, 378–386 (2008). 17621267 10.1038/sj.onc.1210648

[b37] Pignot G. . microRNA expression profile in a large series of bladder tumors: identification of a 3-miRNA signature associated with aggressiveness of muscle-invasive bladder cancer. Int J Cancer. 132, 2479–2491 (2013). 23169479 10.1002/ijc.27949

[b38] Ito S. . Role of Tet proteins in 5 mC to 5 hmC conversion, ES-cell self-renewal and inner cell mass specification. Nature. 466, 1129–1133 (2010). 20639862 10.1038/nature09303 PMC3491567

[b39] Kidoya H. . The apelin/APJ system induced maturation of the tumor vasculature and improves the efficiency of immune therapy. Oncogene. 31, 3254–3264 (2012). 22037214 10.1038/onc.2011.489

