# Supplementary material for: Regulation of SLD5 gene expression by miR-370 during acute growth of cancer cells
Source: Sci Rep. 2016 Aug 8;6:30941. doi: 10.1038/srep30941 (PMC4976388; doi:10.1038/srep30941)
Supplement: Supplementary Information [file srep30941-s1.pdf]

Regulation of SLD5 gene expression by miR-370 during acute growth of cancer cells

Keitaro Yamane, Hisamichi Naito, Taku Wakabayashi, Hironori Yoshida, Fumitaka Muramatsu, Tomohiro Iba, Hiroyasu Kidoya, Nobuyuki Takakura

Department of Signal Transduction, Research Institute for Microbial Diseases, Osaka University, 3-1 Yamada-oka, Suita, Osaka 565-0871, Japan.

FigS.1

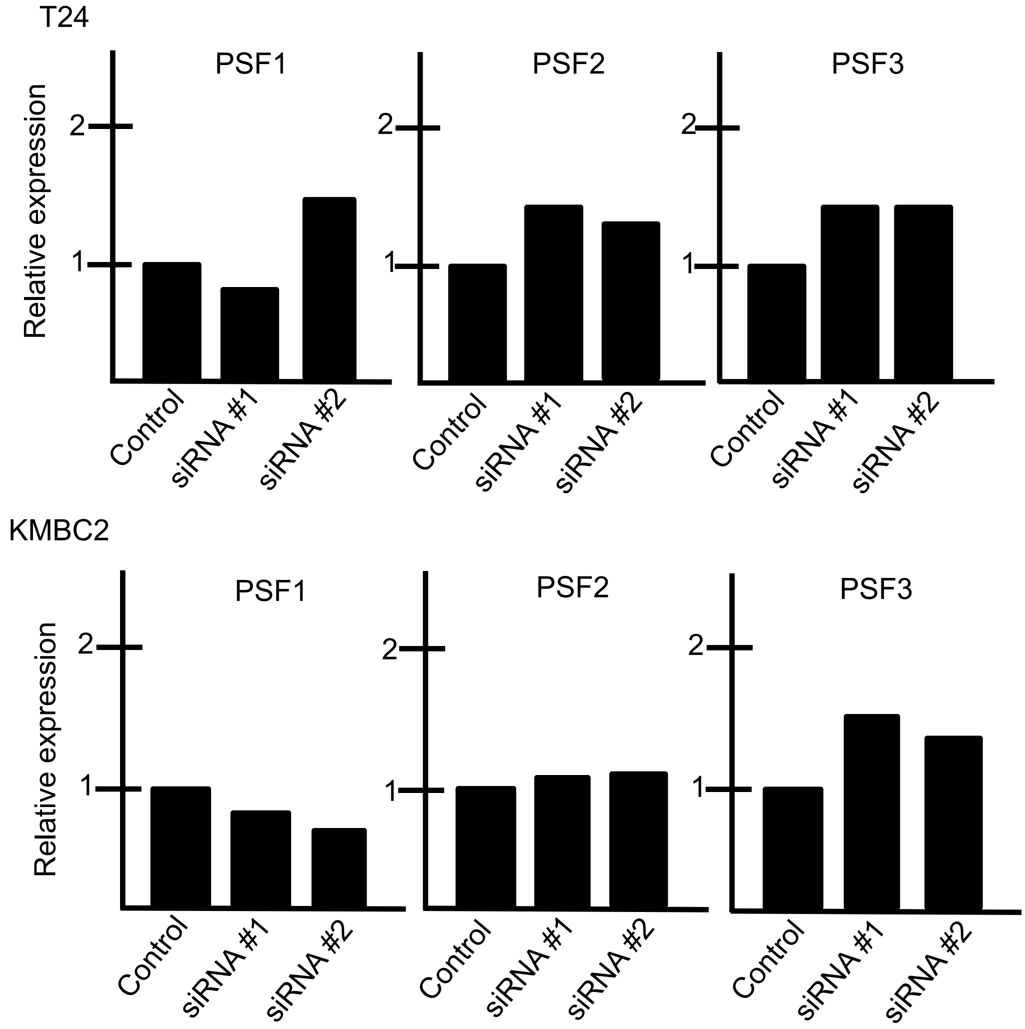

Figure S1. Effect of silencing *SLD5* on mRNA expression of other GINS members.
SLD5 expression was silenced as shown in Fig 2A. qRT-PCR analysiswas performed to detect *PSF1*, *PSF2*, and *PSF3* expression. Note that GINS family genes other than *SLD5* were not affected by the knock down of SLD5 *in vitro* in either T24 or KMBC2 cells. Values for control cells were set at unity.

FigS.2

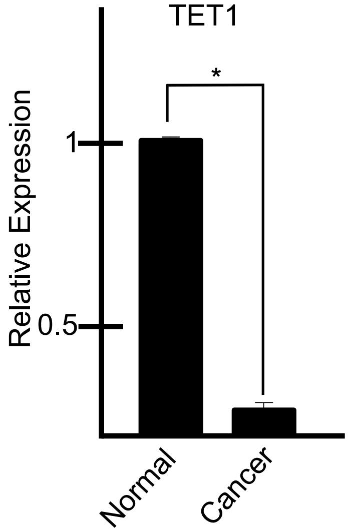

Figure S2. *TET1* expression in cancer cells.
qRT-PCR analysis was performed to detect *TET1* mRNA expression in bladder cancer cell line (T24). Data are mean±SE, ***p<0.0005 (n=3)

FigS.3

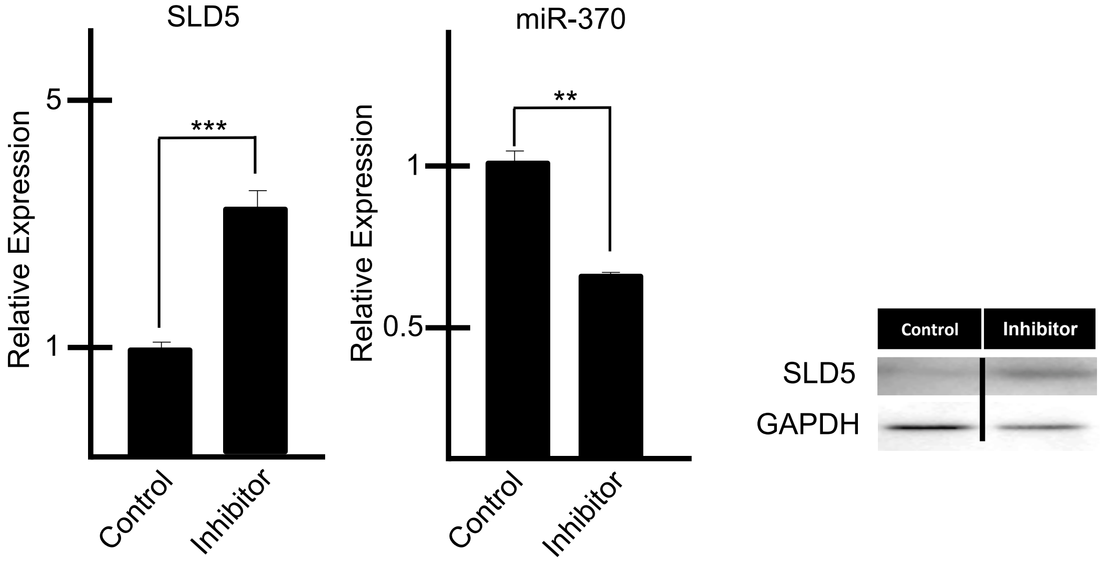

Figure S3. Attenuation of miR-370 expression by miR-370 inhibitor transfection in normal cells. miR-370 expression in HUVEC was inhibited by pre-hsa-miR-370 inhibitor (QIAGEN) under the manufacturer's instructions. After 48h, miRNA and RNA were isolated and cDNA was synthesized for the analysis of miR-370 and *SLD5* respectively. At the same time, protein was also isolated for western blotting analysis of SLD5 expression level. The level of miR-370 (left) and *SLD5* (right) after transfection of miR-370 inhibitor was confirmed by qRT-PCR. Values for control cells were set at unity. *U6* or *GAPDH* was used as an internal control. Data are mean±SE **$p<0.001$, ***$p<0.0001$. (n=3). Western blotting showed that SLD5 expression was increased in miR-370 inhibitor-transfected HUVEC.

Supplementary Table1

Primer set

hSLD5	Fwd	5'-CCAAATGGAGATGGAGAGGA-3'
	Rev	5'-CAGAGAGTTCATGGCGAACA-3'
hPSF1	Fwd	5'-GGTCACTGGGAGGAGATGAA-3'
	Rev	5'-CAGACAAGGAGTCCTGGAGC-3'
hPSF2	Fwd	5'-ACACTCGTATAGCCAAACTC-3'
	Rev	5'-CACTCCTGCTGTATCTACAC-3'
hPSF3	Fwd	5'- GGAGAGGGGCTTATTTCAAA-3'
	Rev	5'-GTGGCTAGTGAAACTGTACA-3'
hIL-6	Fwd	5'-GGTACATCCTCGACGGCATCT-3'
	Rev	5'-GTGCCTCTTTGCTGCTTTCAC-3'
hDNMT1	Fwd	5'-CCATCAGGCATTCTACCA-3'
	Rev	5'-CGTTCTCCTTGTCTTCTCT-3'
hTET1	Fwd	5'-TAAGAAAAGAAAATGTGAGGAGCT G-3
	Rev	5'-CCATTTACTGGTTTGTTGTCAAAA T-3'
hGAPDH	Fwd	5'-CGACCACTTTGTCAAGCTCA-3'
	Rev	5'-CAGTTGCCATGTAGACCCCT-3'
miR-370		5'-GCCTGCTGGGGTGGAACCTGGT-3'
